# Climate change projections for sustainable and healthy cities

**DOI:** 10.5334/bc.111

**Published:** 2021-09-30

**Authors:** Clare Goodess, Sarah Berk, Satyaban Bishoyi Ratna, Oscar Brousse, Mike Davies, Clare Heaviside, Gemma Moore, Helen Pineo

**Affiliations:** Climatic Research Unit, School of Environmental Sciences, University of East Anglia, Norwich, UK; Climatic Research Unit, School of Environmental Sciences, University of East Anglia, Norwich, UK Satyaban Bishoyi Ratna; Climatic Research Unit, School of Environmental Sciences, University of East Anglia, Norwich, UK; The Bartlett School of Environment, Energy and Resources, Faculty of the Built Environment, University College London, London, UK; The Bartlett School of Environment, Energy and Resources, Faculty of the Built Environment, University College London, London, UK; The Bartlett School of Environment, Energy and Resources, Faculty of the Built Environment, University College London, London, UK; The Bartlett School of Environment, Energy and Resources, Faculty of the Built Environment, University College London, London, UK; The Bartlett School of Environment, Energy and Resources, Faculty of the Built Environment, University College London, London, UK

**Keywords:** cities, climate change, climate models, health, local climate zone, planning, projections, urban climate, urban heat island

## Abstract

The ambition to develop sustainable and healthy cities requires city-specific policy and practice founded on a multidisciplinary evidence base, including projections of human-induced climate change. A cascade of climate models of increasing complexity and resolution is reviewed, which provides the basis for constructing climate projections—from global climate models with a typical horizontal resolution of a few hundred kilometres, through regional climate models at 12–50 km to convection-permitting models at 1 km resolution that permit the representation of urban induced climates. Different approaches to modelling the urban heat island (UHI) are also reviewed—focusing on how climate model outputs can be adjusted and coupled with urban canopy models to better represent UHI intensity, its impacts and variability. The latter can be due to changes induced by urbanisation or to climate change itself. City interventions such as greater use of green infrastructure also have an effect on the UHI and can help to reduce adverse health impacts such as heat stress and the mortality associated with increasing heat. Examples for the Complex Urban Systems for Sustainability and Health (CUSSH) partner cities of London, Rennes, Kisumu, Nairobi, Beijing and Ningbo illustrate how cities could potentially make use of more detailed models and projections to develop and evaluate policies and practices targeted at their specific environmental and health priorities.

**Practice Relevance:**

Large-scale climate projections for the coming decades show robust trends in rising air temperatures, including more warm days and nights, and longer/more intense warm spells and heatwaves. This paper describes how more complex and higher resolution regional climate and urban canopy models can be combined with the aim of better understanding and quantifying how these larger scale patterns of change may be modified at the city or finer scale. These modifications may arise due to urbanisation and effects such as the UHI, as well as city interventions such as the greater use of grey and green infrastructures.

There is potential danger in generalising from one city to another—under certain conditions some cities may experience an urban cool island, or little future intensification of the UHI, for example. City-specific, tailored climate projections combined with tailored health impact models contribute to an evidence base that supports built environment professionals, urban planners and policymakers to ensure designs for buildings and urban areas are fit for future climates.

## 1 Introduction

Human-induced climate change is one of the major challenges facing cities in both the Global North and Global South. Many cities are experiencing local changes associated with the 1°C of global warming above pre-industrial levels which has already occurred (Revi *et al*. 2014). Even if international efforts directed at the 2015 Paris Agreement to limit global warming to 1.5°C, cities—as environments holding the majority of the global population—will still need to adapt to inevitable climate change (IPCC 2018). Transformational changes are required over the next few decades to develop sustainable and healthy cities^1^ (Pineo 2020; Rydin *et al*. 2012), *i.e*. cities which enable ‘all people, communities and natural systems to thrive now and into the future’ (Crane *et al*. 2021: 4). These changes will only be effective if they take into account the potential impacts of climate changes at a local scale; particularly changes in the frequency and intensity of extreme weather events such as heatwaves, flooding, drought and storms.

Policy and practice addressing climate change in cities need to encompass actions to enhance resilience and adaptation to climate change as well as to reduce greenhouse gas (GHG) emissions (EEA 2020). The potential co-benefits of adaptation and mitigation also need to be considered, alongside the co-benefits of addressing both climate change and air pollution—particularly with respect to human health (Hess *et al*. 2014).

The demand for a stronger reflection of climate science in built environment health guidance (Pineo 2020) implies a clear and urgent need for appropriate information about climate variability and change tailored for specific cities and for city policy and decision-makers (Bai 2018). This includes a need for improved projections of climate change over the coming decades under different GHG emissions and urbanisation scenarios for individual cities. However, the development of robust and reliable projections for cities at appropriate high-spatial resolutions faces several research gaps and challenges (Bai 2018; Revi *et al*. 2014). The starting point for these projections is the outputs from dynamical global climate models (GCMs) and regional climate models (RCMs).

The urban effects on climate—among which the urban heat island (UHI) is a well-known phenomenon—are, however, not explicitly or routinely simulated in the current generation of these models (Lauwaet *et al*. 2015). This is a particular disadvantage when it comes to the assessment of changes in the risk of heat stress, including the implications for human mortality and comfort (Heaviside *et al*. 2017) because densely populated areas are poorly simulated. This further hampers the study of inequalities in the distribution of the risks of exposure (Hsu *et al*. 2021) and the capacities of actions of people exposed (Venter *et al*. 2020). Studies employing models at resolutions equipped to characterise the urban environment (on the order of 1 km horizontal resolution) have quantified the intensity of the UHI and show that the excess heat directly associated with the UHI could be responsible for up to 50% of total heat-related mortality during heatwaves in a UK city (Heaviside *et al*. 2016). Moreover, the populations exposed to the highest UHI intensities within the city tend to demonstrate higher levels of deprivation (Macintyre *et al*. 2018).

The UHI is a well-known phenomenon in which the presence of an urban area alters the surface energy budget and modifies the surrounding atmosphere, forming an urban boundary layer (Oke 1976, 1978: 273–294). The UHI intensity is generally assumed to be positive, demonstrating higher temperatures in the urban area compared with the surrounding rural area. A recent global review suggests an average UHI intensity of 2°C, reaching 10°C in larger cities and in certain locations and weather conditions (Chapman *et al*. 2017). However, under particular conditions, and often during daytime, an urban cool island (UCI) or negative UHI can develop, particularly in dry and arid climates, or when tall buildings provide shading from sunlight (Bornstein *et al*. 2012).

It is widely assumed that rising temperatures due to anthropogenic climate change will be amplified in cities due to an increase in the intensity of the UHI (Estrada *et al*. 2017). However, if rural temperatures were to rise faster than urban temperatures, the intensity of the UHI would decrease, *e.g*. temperatures at rural reference stations may rise faster than in already heavily urbanised areas due to larger relative changes in the urban fraction in rural areas in recent decades. Some of the first modelling studies that attempted to incorporate the UHI in climate projections indicate that any increase in the average UHI intensity may be limited and, in some cases, might even decrease in relative terms (Eunice Lo *et al*. 2020; Fischer *et al*. 2012; Lauwaet *et al*. 2015; McCarthy *et al*. 2011; Oleson 2012). These studies highlight the need for more detailed modelling focusing on a larger range of cities in different climatic regions. Such simulations need to account for urbanisation and be scaled down to the city and eventually neighbourhood scales (Bai 2018). Modelling approaches are also required that allow the evaluation and exploration of potential interventions for mitigating and adapting to harmful effects of the UHI, including heat stress, such as the use of urban blue and green space and the use of cool or reflective materials. For example, modelling suggests that cool (reflective) and green roofs have the potential to reduce the UHI and the associated heat-related mortality in UK and US cities (He *et al*. 2020; Macintyre & Heaviside 2019; Macintyre *et al*. 2021a).

This paper considers these challenges from the perspective of the Wellcome-funded Complex Urban Systems for Sustainability and Health (CUSSH) project.^2^ CUSSH is a five-year research collaboration aiming to improve capacity to guide transformational health and environmental changes in cities. The programme seeks to promote city transformation for improved environmental quality, sustainability and health by bringing together groups of researchers, decision-makers such as policymakers, public health and built environment professionals, and public groups in the development and use of research evidence (Belesova *et al*. 2018; Moore *et al*. 2021; Zimmermann *et al*. 2020). Six partner cities were selected to represent larger and smaller cities with diversity in income, governance systems, geography and environmental challenges: London (UK) and Rennes (France) in Europe, Nairobi and Kisumu (Kenya) in Africa, and Beijing and Ningbo (China) in Asia (Table 1). These cities are considering a range of different adaptation options or interventions to address climate change as well as wider environmental, social and health-related sustainability issues. Climate information is only one component of the scientific evidence base being developed in the CUSSH project. The actions the CUSSH project aims to promote are based on multi-sectoral policies, addressing challenges at the broader system level encouraging an integrated approach to interventions, which impact upon both human and planetary health (Davies *et al*. 2021).

The multidisciplinary, multi-agency and multi-context approach taken by CUSSH is consistent with the definition of risk adopted by the Intergovernmental Panel on Climate Change (IPCC) since 2012 for its assessment reports (IPCC 2013). In this definition, risk is considered as a function of hazard, exposure and vulnerability. The focus in this paper is on the hazard, *i.e*. climate variability and change including extreme events, particularly those related to heat. This approach is also consistent with the Climate Change Risks and Vulnerabilities reporting template included in the Urban Adaptation Support tool developed to support the Covenant of Mayors for Climate and Energy.^3^ This template facilitates reporting of current risks of hazards (probability and impact) and future hazards (changes in intensity and frequency, and timeframe) including climate hazards: extreme heat, extreme cold, heavy precipitation, floods and sea level rise, droughts and water scarcity, and storms. The next step of assessing issues such as the health risks associated with an increase in extreme heat requires a consideration of a complex mix of socio-economic vulnerabilities and built environment characteristics which are beyond the scope of this paper (Ellena *et al*. 2020; Heaviside *et al*. 2017; Macintyre *et al*. 2018). Major US cities, for example, experience substantial intra-city variations in heat exposure risk that represent income and racial inequalities (Hsu *et al*. 2021).

**Figure 1:**
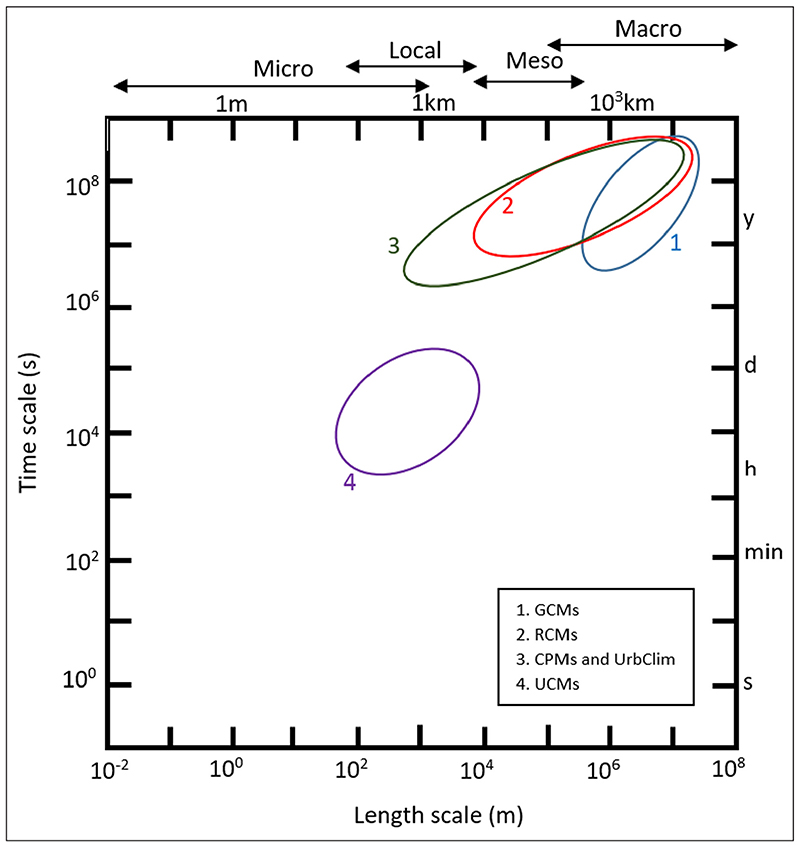
Scales of climate modelling and urban climate components.

This paper is focused on projections of climate change over the next few decades, based on different pathways of GHG concentrations. The focus is, therefore, more on changes in average climate (typically over 20–30 years) and the UHI than day-to-day or year-to-year variations. Consideration is given, however, to changes in extreme events, particularly those related to heat. There is some mismatch between the spatial scales of the climate models used to construct these projections and microscale models which incorporate urban canyon and other such effects and are typically run for considerably shorter periods. This paper uses the scale definitions shown in Figure 1, which reflect the different scales of urban phenomena (Grimmond *et al*. 2010; Oke 2006). These include the urban canopy layer at the microscale, the surface urban boundary layer at the local scale and the outer urban boundary layer at the mesoscale (see File 1 in the supplemental data online).

The focus of this paper is more on the city-wide scale (meso- to macroscale) and broad variations across a city, rather than presenting quantitative projections at the neighbourhood or smaller scale (local to microscale). Nonetheless, potential methodologies as to how such city-wide projections can be further downscaled or used in conjunction with higher resolution information are discussed.

The paper is structured as follows. The general characteristics of the six CUSSH partner cities, including their different environmental and health priorities, are outlined and compared in Section 2. Section 3 outlines the contributory causes of the UHI and their relationship with potential measures to reduce its intensity and impacts. A cascade of climate models of increasing complexity and spatial resolution is described in Section 4, together with models for incorporating urban canopy processes in these models. GCM-based projections for the CUSSH partner city-regions are presented in Section 5, and potential methods for better tailoring these projections to the needs of CUSSH city stakeholders and decision-making outlined in Section 6. The concluding remarks in Section 7 focus on interdisciplinary linkages between climate experts and experts in urban health, governance and planning, and engagement with users of climate information in cities.

## 2 The Cussh Partner Cities

The six CUSSH partner cities were selected to represent cities with different socio-political, geographical, environmental and city-size contexts, all with stakeholders willing to engage in participatory work with the CUSSH research partners (Belesova *et al*. 2018; Moore *et al*. 2021; Zimmermann *et al*. 2020). The general characteristics of these cities are summarised in Table 1.

Four of the cities are located in Northern Hemisphere mid-latitudes (London, Rennes, Beijing and Ningbo) and two are situated in the equatorial region (Kisumu and Nairobi). The cities range from small (Rennes and Kisumu, each with fewer than 350,000 inhabitants) to megacity (Beijing with close to 20 million inhabitants) size. The only truly coastal city is Ningbo on the East China Sea, though London is located at the upper end of the Thames River estuary and Kisumu is a port city on Lake Victoria.

In terms of general climate characteristics, London and Rennes are broadly similar, both classified as Temperate (Table 1). Rennes is somewhat warmer and wetter than London. Of the two Chinese cities, Beijing is colder and considerably drier than Ningbo, reflecting its continental location. Kisumu has a clearly Tropical climate and is both the warmest and wettest of the six cities. Nairobi lies in a region of spatially variable climate, but is the second warmest of the six cities, and has some characteristics of a Tropical climate. It is not the purpose of this paper to provide a detailed description of the climate or weather of each city, but it is noted that moisture availability in the surrounding rural area as well as in the city itself will have an influence on the intensity of the UHI. The UHI is expected to reach a maximum during the daytime and wet season in some Tropical climates, with potential for a UCI to form during the dry season (Bornstein *et al*. 2012). In contrast, the UHI is expected to be strongest at night in Temperate climates such as those of London and Rennes (Kolokotroni & Giridharan 2008).

The observed UHI has been relatively well studied for cities in China (Zhou *et al*. 2015) and for London (Jones & Lister 2009; Zhou *et al*. 2016) using both urban/rural station pairs (which measure the air temperature in the canopy layer) and satellite data (which measure the surface UHI). Estimates of the UHI based on station data are very sensitive to the station location and it can be difficult to identify truly urban and rural stations (Heaviside *et al*. 2017). Studies for London indicate, however, that the average central London UHI, which is in the order of 0.6– 0.9°C for maximum temperature and 1.6–2.8°C for minimum temperature, likely developed and stabilised before the start of the 20th century (Jones & Lister 2009). In contrast, rapid ongoing urbanisation in cities such as Beijing means continuing enhancement rather than stagnation of the UHI (Ren *et al*. 2007).

As well as differences in geography and climate, the relevant planning and environmental governance systems are different across the CUSSH partner cities (Table 1), which requires consideration in the research and modelling process if outputs are to be useful. For instance, an analysis of the use of evidence in urban health and sustainability policymaking in the Chinese CUSSH partner cities demonstrated the importance of national policy and extensive use of officially commissioned environmental health research and monitoring indicators (Pineo *et al*. 2021). The environmental governance practices in Beijing and Ningbo are comparatively stronger with regard to the use of such data in policymaking, demonstrating the importance of understanding what Oliver & Boaz (2019) call ‘cultures of evidence use’. Similarly, to increase the use of models about the health effects of climate mitigation, Hess *et al*. (2020) advocate stakeholder engagement with policymakers. The need to engage end users in modelling processes recognises that achieving effective change is contingent upon the active involvement of city decision-makers, planners and other actors—formal and informal—who affect implementation (Moore *et al*. 2021). Corburn (2009) provides one such example, describing a co-production framework to ‘localise’ climate research of global climate scientists working with city planners to devise contextually relevant strategies to address the UHI. It is frequently assumed that there is a rational and linear process for translating evidence to action (or theory to practice); however, the role of systems, relationships, different forms of knowledge and contexts within such processes are key. There is increasing recognition of the complex and contested nature of policy processes and the use of evidence within these processes (Pineo *et al*. 2020).

For CUSSH, these complexities of city governance and planning are taken into account in the project protocol (Davies *et al*. 2021) and programme theory (Moore *et al*. 2021). Participatory methods have been used to work with stakeholders in each CUSSH partner city to identify the preliminary environmental and health priorities for the research (Belesova *et al*. 2018; Zimmermann *et al*. 2020). These priorities are listed in Table 1 and their implications for the provision and use of climate information discussed in Section 6.

## 3 Contributory Causes of The Uhi and City Interventions

Present-day impacts and the future evolution of the UHI are of concern for most, if not all, of the CUSSH partner cities (see Sections 2 and 6). Six factors that affect the surface energy balance of a city, and are therefore contributory causes of the UHI, have been identified (Oke 1981). These are listed below and described in File 1 in the supplemental data online:

- anthropogenic heat
- impervious surfaces
- thermal properties of the city fabric
- surface geometry
- urban roughness
- air pollution.

Many of the climate change adaptation and mitigation measures that are available to cities (EEA 2020), including many of those identified in the RESIN Adaptation Options Library,^4^ have the potential to affect these six factors contributing to the UHI and to moderate its extent and magnitude (Fallmann & Emeis 2020; Heaviside *et al*. 2017; Mirzaei 2015). The range of interventions being deployed by city planners and policymakers worldwide includes those being explored in order to address the environmental and health priorities of the CUSSH partner cities (see Table 1 and Section 6). A switch from private cars to active travel, as is being promoted in London and Rennes, for example, will help to reduce anthropogenic heat. The successful implementation of ambitious air pollution targets as seen recently in Chinese cities, including Beijing and Ningbo, could reduce longwave radiation and thus the nocturnal surface UHI—though such relationships are complex.

The use of shading and cool materials that improve reflectivity of roofs, roads and other surfaces or reduce the thermal absorption of surfaces are examples of grey infrastructure which can modify the thermal properties of buildings and cities (Krayenhoff *et al*. 2018). The use of green infrastructure, particularly blue and green space including parks and roofs, is a nature-based intervention being considered and adopted by many cities worldwide (Estrada *et al*. 2017), including London (Zimmermann *et al*. 2020). The associated changes in thermal properties of the city fabric, impervious surfaces and evapotranspiration have the potential to reduce UHI intensity and heat-related mortality (Gunawardena *et al*. 2017; He *et al*. 2020; Macintyre & Heaviside 2019; Macintyre *et al*. 2021a; Mirzaei 2015; Reder *et al*. 2018).

The next section considers the extent to which these urban processes that underpin the efficacy of different interventions can be incorporated in the climate models used to explore the impacts of anthropogenic climate change.

## 4 Modelling Approaches for City Climate Projections

### 4.1 Global Climate Models (GCMs)

The standard starting point for developing projections of climate change over the coming decades are GCMs or increasingly Earth system models (ESMs) (Collins *et al*. 2013; Flato *et al*. 2013). These numerical models simulate the well-documented physical processes that control the transfer of energy and materials through the climate system, and which cause the large-scale patterns of atmospheric and ocean circulation that drive weather and climate. They use equations and parameterisations at the grid-box level (*e.g*. spatial resolution of a few 100 km) (Figure 1) to represent these processes and interactions, encompassing the major components of the climate system (atmosphere, land surface, ocean and sea ice). Internationally coordinated GCM simulations (Coupled Model Intercomparison Project—CMIP), forced by standard GHG emissions scenarios, provide a key input to the IPCC assessment reports. The large multi-model CMIP Phase 5 (CMIP5) ensemble (Taylor et al. 2012) forced by representative concentration pathways (RCPs) (van Vuuren *et al*. 2011) was developed ahead of the IPCC’s *5th Assessment Report* (IPCC 2013) and CMIP Phase 6 (CMIP6) features strongly in the *6th Assessment Report* (IPCC 2021).

Whilst GCM outputs such as those from CMIP5 have been used to develop consistent climate projections for multiple cities (Guerreiro *et al*. 2018), including projections for the CUSSH city-regions (see Section 5), urban areas and the processes that cause the UHI (see Section 3) are not simulated in these models (Lauwaet *et al*. 2015). Attempts have been made to incorporate relatively simple urban canyon models into the land component of GCMs (Fischer *et al*. 2012; Oleson 2012), but the rather coarse spatial scale of the GCMs used for these studies (about 1° latitude/longitude) limits the utility of such simulations for UHI studies. In a more recent sensitivity study, an urban canyon representation scheme at a global resolution of 50 km has been embedded into a GCM to explore the global impacts of cities on climate as well as the effects of climate change on the UHI (Katzfey *et al*. 2020).

### 4.2 Regional Climate Models (RCMs)

For many assessments of the impacts of climate change, including impacts in urban areas, there is a need to downscale to finer spatial resolutions than those conventionally provided by GCMs and ESMs. Dynamical downscaling is performed using RCMs that employ the same equations and parameterisations as GCMs, but which are run at higher spatial resolutions (Flato *et al*. 2013). RCM simulations are performed over smaller domains nested within GCMs, *i.e*. GCM outputs are used to provide boundary conditions for the RCMs (Giorgi 2019). Multi-model RCM ensembles of varying size have been produced for domains covering most of the globe, including Europe (Jacob *et al*. 2014), Africa (Nikulin *et al*. 2018) and East Asia (Niu *et al*. 2018) as part of the Co-Ordinated Regional Downscaling Experiment (CORDEX).^5^ The standard CORDEX simulations are forced by outputs from CMIP5 GCMs and have a grid-box resolution of between 50 km (0.44° latitude/longitude) and 12 km (0.11° latitude/longitude), with outputs freely available. The CORDEX and other RCM simulations are widely used to explore the impacts of climate change, including those in cities (Abiodun *et al*. 2017; Eunice Lo *et al*. 2020).

While the higher spatial resolution of RCMs (Figure 1) can add value, particularly in topographically complex regions such as coastal and mountain regions (Giorgi 2019; Rummukainen 2016), the standard RCM simulations, such as with GCMs, still do not include explicit representations of urban areas or processes. In an early study conducted to address this gap, McCarthy *et al*. (2011) coupled a 25 km resolution RCM with a simple land-surface exchange scheme including an urban tiling scheme and heat emissions to develop city-scale projections of the UHI for the UK. Offline simulations using more sophisticated and higher resolution (*e.g*. 250 m) land surface schemes and urban canopy models (UCMs) such as the Town Energy Balance (TEB) model provide improved information about the impact of built-up surfaces on land-atmosphere interactions (*e.g*. Roberge & Sushama 2018). In a more recent coupled model sensitivity study, the TEB model has been run with the ALADIN RCM at 12 km to demonstrate the benefits for simulating the UHI of large cities in France (Daniel *et al*. 2019). Additionally, Krayenhoff *et al*. (2018) used the Single Layer Urban Canopy Model (SLUCM) (Kusaka *et al*. 2001) embedded in the Weather Research and Forecast (WRF) RCM to quantify the effect of the expected urban expansion of US cities under an RCP8.5 scenario. This study also tested the effect of different city interventions (adaptation strategies) and suggested their effect to be more pronounced during afternoons of extreme hot days.

### 4.3 Convection-Permitting Models (CPMs)

Even at 12 km resolution, important local-scale and sub-daily processes such as intense convective storms which can lead to flash flooding particularly in urban areas are still not explicitly resolved in RCMs. Thus, there is increasing interest in using CPMs for developing climate projections (Prein *et al*. 2015). CPMs are typically based on the numerical weather prediction models used for weather forecasting and have a spatial resolution of 1–4 km (Figure 1). In general, CPMs, are necessary to run UCMs of varying complexity. To better model UHIs of specific cities, choosing the right CPM is important (Argüeso *et al*. 2014). Using CPMs as a tool to further downscale RCM simulations gives the potential to develop city-scale climate projections and test adaptation strategies (city interventions) for the present day and future at 1–2 km resolution (*e.g*. Argüeso *et al*. 2014; Brousse *et al*. 2016; Kendon *et al*. 2019; Oleson *et al*. 2015; Wouters *et al*. 2017).

### 4.4 Urban Canopy Models (UCMs)

The urban surface schemes used with GCMs, RCMs and CPMs can be split into three groups: bulk parameterisation, single-level UCMs and multilevel UCMs. In bulk parameterisation, the urban surface is represented by bare soil or a flat plate (a slab) with modified roughness length and thermal properties. There is reduced moisture availability to ensure sensible heat fluxes are favoured over latent (Best 2005).

Single-layer UCMs add more complexity. They represent the general characteristics of urban morphology, but do not take into account microscale aspects such as individual buildings or parks (*e.g*. Best 2005; Masson 2000; Reder *et al*. 2018). The most complex models are multilevel UCMs. These provide a more detailed representation of the urban form, dividing surfaces into several horizontal patches with their own energy balances (Grimmond *et al*. 2010).

The choice of parameterisation depends on the goal of the study. Multilevel UCMs are useful for studying complex urban interactions (Best 2005; Chen *et al*. 2011), but are computationally expensive and require detailed input values (Demuzere *et al*. 2017; Zonato *et al*. 2020). Simpler approaches are useful for longer runs, as needed in the context of climate projections, and in an international comparison project have been found to perform as well as more complex schemes (Grimmond *et al*. 2010) and even to better represent the seasonal cycle of the UHI (Best & Grimmond 2013).

Thus, bulk parameterisations such as TERRA-URB urban land-surface scheme (Wouters *et al*. 2016) or single-layer UCMs such as TEB (Masson 2000) and SLUCM (Kusaka *et al*. 2001) are a popular choice for use with RCMs (Daniel *et al*. 2019; Roberge & Sushama 2018; Wouters *et al*. 2017). TEB and SLUCM consider three surfaces (roofs, roads and walls) and the influence of shadowing and reflection of radiation due to canyon geometry, while TERRA_URB adapts physical parameters of the urban land surface at the bulk level via the semi-empirical urban canopy parameterisation (SURY). Other more computationally efficient approaches that do not use RCMs can also be adopted. For instance, the UrbClim urban boundary layer climate model, which was developed specifically to produce urban climate projections based on CMIP5 GCMs (Lauwaet *et al*. 2015), uses a bulk parameterisation land surface scheme and a simple three-dimensional model of the lower atmosphere. It has been used to produce climate projections for 100 European cities, including London (De Ridder *et al*. 2015), which are made available through the Urban Adaptation Map Viewer,^6^ part of the European Climate Adapt platform.

Multilayer UCMs such as the Building Effect Parameterisation (BEP) model (Martilli *et al*. 2002) reflect the three-dimensional nature of the urban surface and allow vertical exchanges through the urban canopy layer and interactions with the planetary boundary layer (Chen *et al*. 2011). BEP is typically implemented in parallel with the Building Energy Model (BEM) (Salamanca *et al*. 2011), which permits simulation of indoor–outdoor energy exchanges. Simulations performed for Madrid (Spain) demonstrated the utility of integrating BEP–BEM with WRF to quantify the effect of indoor–outdoor exchanges, and of air-conditioning systems in particular, on the urban climate (Salamanca *et al*. 2012). This triggered the coupling of BEM to multiple other UCMs embedded in other RCMs than WRF (*e.g*. Bueno *et al*. 2012; Jin *et al*. 2020).

In parallel, Brousse *et al*. (2016) showed over Madrid that together with local climate zone (LCZ) typologies to partition the urban area to the neighbourhood scale, *i.e*. about 350 m, UCMs such as BEP–BEM can efficiently be parameterised for any city a cross the globe. In short, the use of multiple LCZs allows classification of individual cities into multiple urban categories with different morphologies, building types and densities, and allows better representation of the differences between individual cities (Stewart & Oke 2012). This approach allows differentiation between, for example, the ultra-dense and compact cities with high-rise buildings of China and the sprawling and low-density cities with low-rise residential buildings more typical of North America and Australia. Thanks to the World Urban Database and Access Portal Tool (WUDAPT) project,^7^ LCZ can easily be mapped following a standardised procedure (Ching *et al*. 2018). Multiple urban climate modelling studies have now used LCZ to parameterise UCMs in a variety of RCMs and model the urban climate of major cities across the globe (*e.g*. Alexander *et al*. 2016; Brousse *et al*. 2020; Hammerberg *et al*. 2018, Varentsov *et al*. 2020; Verdonck *et al*. 2018; Wong *et al*. 2019).

### 4.5 A Modelling Toolbox for Cities

A cascade of dynamic climate models of increasing spatial scale (Figure 1) suitable for developing climate projections for cities has been presented in Sections 4.1–4.3. A similar cascade of UCMs of increasing complexity is identified in Section 4.4. Each of these models has particular advantages and disadvantages. Increased complexity and resolution come at the cost of increased computational intensity, and no one type of model can answer all the issues and questions that need to be addressed by cities tackling climate change.

In addition to inevitable uncertainty concerning which emissions scenario society will follow (van Vuuren *et al*. 2011), there are inherent climate modelling uncertainties associated with the response of the climate models to greenhouse forcing. It is good practice to use several models from different modelling centres—such as those provided by the CMIP5 GCM ensemble. This ensemble also has the advantage of providing information based on a common set of models for multiple cities and emissions scenarios (Guerreiro *et al*. 2018; Revi *et al*. 2014). GCM-based projections for the CUSSH city-regions are presented in Section 5.

This review has identified some of the promising approaches for coupling high-resolution climate and urban models in order to better understand issues such as changes in the UHI and the implications for health (Heaviside *et al*. 2017; Wouters *et al*. 2017). Many of the studies undertaken to date are exploratory in nature, and due to their high computational demands are limited in terms of the number and type of cities considered, as well as length of the simulations and application to multiple climate models and emissions scenarios. Section 6 discusses the potential for applying these emerging approaches to the CUSSH cities.

## 5 Climate Projections for The Cussh City-Regions

In order to provide climate projection data for the regions containing each of the partner cities during the early stages of CUSSH, use was made of a large pre-existing data set. This is the post-processed CMIP5 ensemble of GCM runs (see Section 4.1) used to produce country profiles^8^ for the World Health Organization (WHO). The original data set produced for WHO included annual mean temperature and precipitation totals (and derived indices of extremes calculated from daily data; see Table S1 in the supplemental data online), for a low and a high emissions scenario: RCP2.6 and RCP8.5, respectively (van Vuuren *et al*. 2011) for the period 1900–2100.

One of the main advantages of the CMIP5 data is the relatively large ensemble size—important for assessing climate modelling uncertainty (about 20 GCMs for the WHO analysis)—and the availability of an emissions scenario consistent with the 2°C Paris policy target (RCP2.6), as well as a high non-mitigation scenario (RCP8.5). For CUSSH, an important characteristic of these GCM data and the accompanying gridded observations is that the information base is consistent across cities. For this reason, an earlier version of the WHO data set was used to provide projection data for 246 global cities included in the Sustainable Healthy Urban Environments (SHUE) database (Milner *et al*. 2017). The disadvantage is the relatively coarse spatial scale: the GCMs have a grid-box resolution of a few hundred kilometres (Figure 1).

Six indices of temperature extremes and four indices of precipitation extremes were provided, together with average mean, minimum and maximum temperatures and total annual rainfall. All indices are calculated annually using daily data, *i.e*. a single value is calculated for each year. Indices based on gridded observations^9^ are provided with the projection data. For further details of the data processing, see File 2 in the supplemental data online.

**Figure 2:**
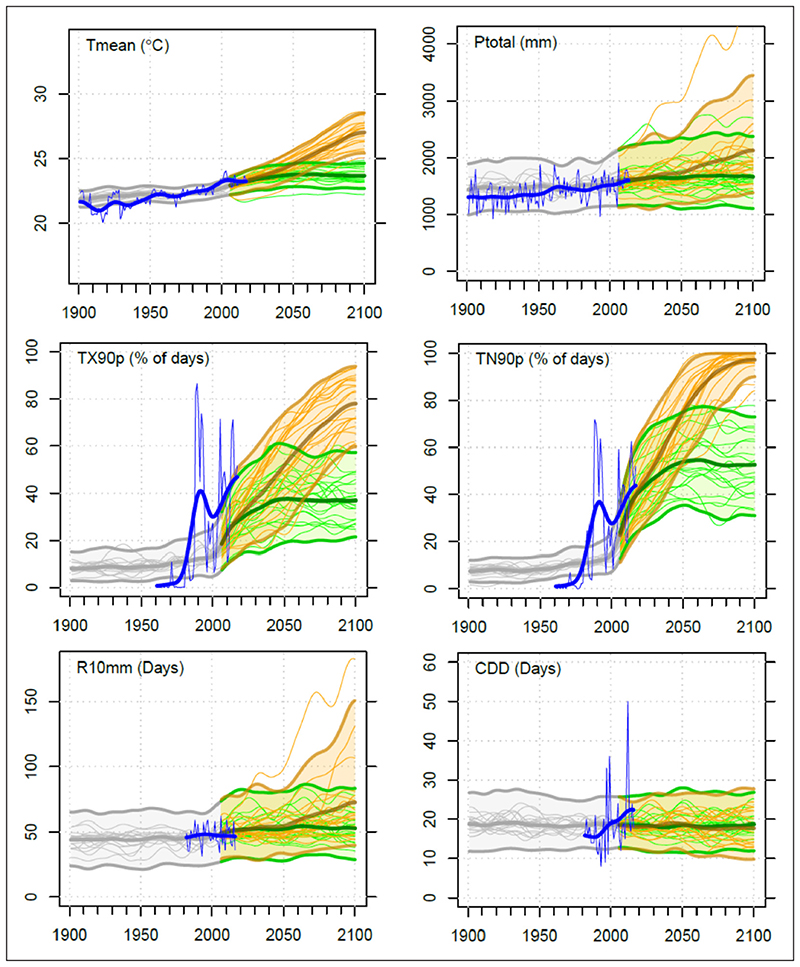
Global climate model (GCM)-based projections for Kisumu. *Note:* These time series show simulated changes across 20 different GCMs under a high emissions scenario (RCP8.5; orange) compared with a scenario with rapidly reducing emissions (RCP2.6; green). The multi-model mean is shown (thick lines) together with the individual models (thin lines), as well as the 90% model range (shaded) as a measure of uncertainty. Observations (smoothed and unsmoothed) are shown in blue. Mean temperature (*T*_mean_; °C), total rainfall (*P*_total_; mm), warm days (TX90p; percentage of days), warm nights (TN90p; percentage of days), heavy rainfall days (R10mm; days) and consecutive dry days (CDD). See Table S1 in the supplemental data online for definitions of the climate indices.

An example of some of the indices produced for Kisumu is shown in Figure 2. All time-series plots show indices for a high emissions scenario (RCP8.5) and a low emissions scenario (RCP2.6). The plots also show each model individually as well as the 90% model range as a measure of uncertainty, together with the annual and smoothed observed record. Summary statistics for Kisumu are presented in Table 2 (observations and projected changes for 30-year time periods). For summary tables and a full set of plots for all six CUSSH cities, see File 2 in the supplemental data online.

The projections for all six cities show clear and consistent trends towards higher mean temperatures and more frequent warm days and nights and fewer cold days and nights, together with longer warm spells and shorter cold spells. For many of the cities, including Kisumu (Table 2), the projected increases in warm nights are considerably larger than those in warm days, indicating greater night-time heat stress. The temperature increases are clearly lower for RCP2.6 than for RCP8.5, but the increases in temperature extremes are still quite large for cities such as Kisumu (*e.g*. a 38% increase in warm nights for the 2080s for RCP2.6, with an uncertainty range of +25% to +54%, compared with an 80% increase for RCP8.5, with an uncertainty range of +67% to +85%) (Table 2).

The projections for rainfall are more uncertain than those for temperature. It is generally difficult to distinguish any change associated with RCP2.6 from natural variability (*i.e*. the ensemble range and year-to-year variability in the observations). For RCP8.5, the general tendency for all cities, apart from Rennes, is for mean annual total rainfall to increase. The largest increase for the 2080s is for Kisumu (+29%, with an uncertainty range of +2% to +76%) and the smallest for London (+4%, with an uncertainty range of –7% to +15%). All cities except Ningbo indicate more intense and frequent rainfall extremes, including an increase in the number of heavy rainfall days. Cities for which little change in total precipitation is projected may still experience an increase in extreme rainfall. In general, these increases are fairly small, reaching a maximum for Kisumu in the case of heavy rainfall days (Table 2). The number of consecutive dry days generally shows little or very uncertain change, though with some tendency to increase slightly for London (a maximum of +8 days with an uncertainty range of 0–14 days for RCP8.5 in the 2080s) and Rennes.

These projections provide a large-scale picture of how the climate—including extremes of temperature and rainfall—of the CUSSH cities may change over the next decades. Such projections need to be interpreted cautiously since they are based on a single grid point extracted from relatively coarse-scale climate models (Figure 1) that do not resolve the cities themselves or urban climates in general and may not well represent regional effects such as the influence of Lake Victoria and the East African rift on the climate of Kisumu (Van de Walle *et al*. 2020). Hence, they are referred to as ‘city-region’ rather than ‘city’ projections. They are presented for annual values only, which may mask differential changes at the seasonal or monthly level, particularly in rainfall (*e.g*. different changes in the short and long rains in Kenya, and summer drying for London and Rennes). Section 6 considers the utility of such projections for CUSSH, as well as how they can be further refined and tailored for specific cities, including consideration of the UHI and city interventions.

## 6 Using Climate Projections in CUSSH

The city-region projections developed for the CUSSH cities provide the basis for a qualitative description of potential changes in climate under two different emissions scenarios (see Section 5). These GCM-based projections are valuable for awareness-raising and highlighting the benefits of climate change mitigation (*i.e*. RCP2.6 compared with RCP8.5). The projections for Kisumu and Nairobi were used to produce climate change risk profiles (two-page briefing notes) for use in participatory workshops with city stakeholders (Belesova *et al*. 2018).

For Kisumu (Table 2 and Figure 2), the potential impacts and risks associated with projected increases in high temperature extremes include:

- human heat stress and other negative health effects including potential increases in mortality
- negative impacts and constraints on labour productivity, particularly for outdoor workers
- greater heat stress and discomfort for residents and tourists, leading to potential increased demand for air conditioning, which would increase energy demand.

Risks associated with the higher annual rainfall totals and more frequent/intense heavy rainfall events projected for Kisumu include:

- increased surface erosion and runoff, with a potential increase in flood risk, particularly where urban and transport developments lead to an increase in non-permeable surfaces
- increased risks to transport infrastructure
- possible implications for water quality and sanitation.

Sanitation issues associated with present-day flooding were raised by city stakeholders in Kisumu focus groups organised by CUSSH (Salvia *et al*., forthcoming). Flooding is reported to be caused by drainage systems blocked by improperly disposed waste, for example, which creates health issues for children, in particular. These issues would be exacerbated by any increase in rainfall, particularly heavy rainfall. Thus, the climate projections support the need for early action by the city to address waste management issues and to avoid future increases in health risks associated with flooding.

These city-region projections (see Section 5) indicate the expected direction of change and highlight why it is important to include climate information in the evidence base for assessing the efficacy of proposed interventions to address the different environmental and health priorities for the CUSSH cities (Table 1). To be effective over the coming decades, green and/or blue space (a priority for London), for example, should be designed to be resilient to a changing climate. In selecting the most appropriate tree species to plant in London currently, for example, consideration should be given to their ability to cope with hotter conditions (Eunice Lo *et al*. 2020), as well as more variable rainfall and generally drier summers (Jacob *et al*. 2014). Similarly, green roofs and drainage systems need to be designed to cope with projected increases in rainfall intensity during convective storms (Kendon *et al*. 2019). The uptake of active travel (a priority for London and Rennes) may be sensitive to increasing heat stress and changes in the frequency and intensity of heavy rainfall. Seasonality should also be considered for adaptation measures such as cool roofs, which are designed to reduce health impacts primarily in warm seasons (Macintyre *et al*. 2021a, 2021b).

The city-region projections do not directly integrate the urban climate anomalies, but they do allow a rapid assessment of potentially suitable city interventions. In order to properly assess the efficacy of implementing these potential solutions or city interventions, more detailed and quantitative modelling of their health impacts is required, as well as more reliable estimates of the magnitude of the changes in climate. The CUSSH research partners are refining and tailoring a number of different impact modelling approaches, including microsimulation combined with a building physics metamodel for assessing outdoor/indoor exposure to air pollution and thermal comfort (Li *et al*. 2019; Symonds *et al*. 2019), the Cities Rapid Assessment Framework for Transformation (CRAFT) for assessing health impacts of policy interventions (Symonds *et al*. 2020), the Health-Oriented Transportation (HOT) model for active travel,^10^ and the Greenhouse Gas–Air Pollution and Synergies (GAINS)^11^ model for quantification of the co-benefits of air pollution and climate change mitigation.

Several of these health impact models would require modification in order to incorporate present-day and future climate conditions. Others require downscaling of climate inputs to a 1 km resolution, or processing into very specific formats such as the weather files used by the EnergyPlus buildings model (Li *et al*. 2019). Methods are available for producing design summer years and other weather files for simulating overheating risk in London buildings using a weather generator, but it would be time-consuming to apply these methods to other locations and scenarios, and availability of appropriate high-temporal resolution observed data could be a limitation.

The spatial scale of CPMs (see Section 4.3) is consistent with the requirements for high-resolution inputs for health impact models. The utility of the new national UK Climate Projections (UKCP) (part of UKCP18; Kendon *et al*. 2019) for CUSSH work in London is currently being explored. The underlying CPM simulations were performed at 2.2 km resolution using the new Met Office Reading Urban Surface Exchange Scheme (MORUSES). This scheme includes two urban tiles representing roofs and street canyons. Surface parameters are determined from the morphology and materials properties of relevant cities (Porson *et al*. 2010). MORUSES is considered to provide a better representation of the urban surface energy balance than the simpler one-tile urban scheme used in the driving 12 km RCM (Kendon *et al*. 2019). Due to the computational intensity, UKCP Local projections are only available for 20-year time slices (1981–2000, 2021–40 and 2061– 80) and for RCP8.5.

**Figure 3:**
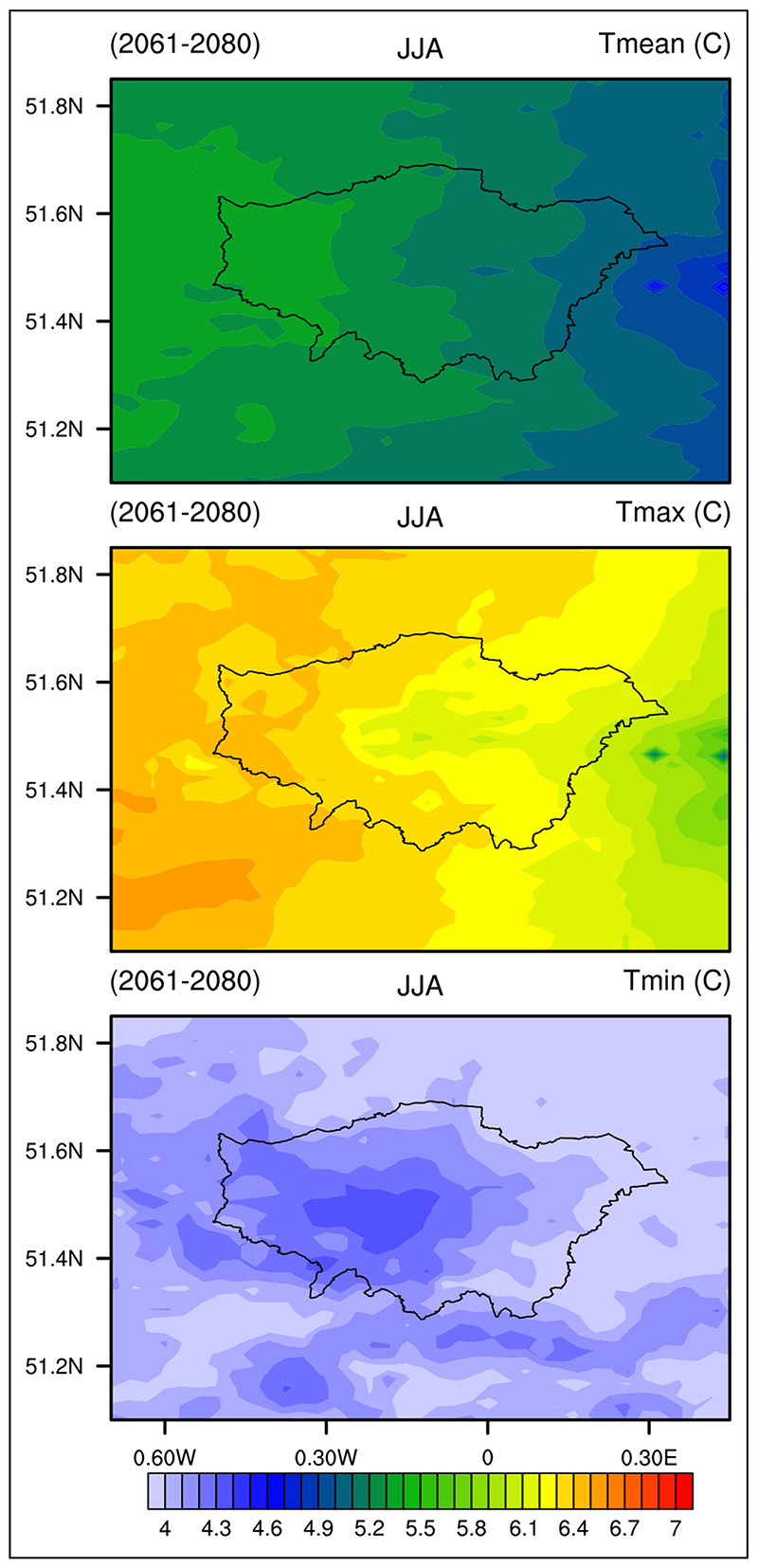
Projected changes (°C) in average (*T*_mean_), maximum (*T*_max_) and minimum (*T*_min_) summer (June–August) temperature across the London area for the period 2061–80 with respect to the period 1981-2000 for RCP8.5. *Source:* UK Climate Projections (UKCP) Local convection–permitting model (CPM)-based simulations: average of the 12-member ensemble.

Figure 3 shows projected changes in average, maximum and minimum summer temperatures over the London area for the period 2061–80 with respect to the baseline period 1981–2000. The changes in average and maximum temperatures appear to reflect the larger scale pattern of warming over the UK seen in both the RCM and CPM (Kendon *et al*. 2019). This warming reaches a maximum in Southern England and is somewhat lower in coastal regions, such as to the east of London. There is, however, an indication of enhanced warming of minimum temperature (which can be considered as the night-time temperature) over the Central London region (Figure 3). Some enhancement of the nocturnal UHI is also seen in the driving 12 km RCM simulations (Eunice Lo *et al*. 2020). This finding differs from earlier studies with coarser and simpler models that indicate little or no change in the average London UHI (Lauwaet *et al*. 2015; McCarthy *et al*. 2011). Further work is needed to determine whether this apparent increase in the intensity of the nocturnal London UHI is robust or if it might be related to model errors such as excessive summer drying in the CPM (Kendon *et al*. 2019).

As part of the collaboration between the Wellcome-funded CUSSH and Health and Economic impacts of Reducing Overheating in Cities (HEROIC) projects,^12^ simulations will be run with the WRF model at 1 km resolution (see Section 4.3) for some of the CUSSH cities—most likely London, Nairobi and Kisumu. The BEP-BEM UCM parameterised by 17 LCZs (see Section 4.4) will be used, building on previous work in Birmingham (UK), Madrid and Barcelona (Spain), Vienna (Austria), Bologna (Italy), Kampala (Uganda) (Brousse *et al*. 2016, 2020; Hammerberg *et al*. 2018; Heaviside *et al*. 2015; Ribeiro *et al*. 2021; Zonato *et al*. 2020). This type of modelling at 1 km resolution can be combined with projections from GCMs/RCMs to investigate the impacts of changes to urban infrastructure and climate change on heat-related mortality (Heaviside *et al*. 2016). Modification of the UCM and LCZ input variables, such as the fraction of roofs/canopy/green surfaces in each grid box, will allow evaluation of specific interventions targeted at reducing the UHI such as increased use of green infrastructure (Mirzaei 2015). In the case of London, this will allow quantification of the qualitative relationship between enhanced use of green infrastructure, health and the UHI identified in system dynamics work with London stakeholders (Zimmermann *et al*. 2020). Evidence derived from HEROIC modelling will be used to inform tools such as CRAFT, *e.g*. for assessing the likely impacts of urban heat reduction through measures such as cool roofs.

Downscaled climate projections will be developed for CUSSH by forcing the 1 km grid-resolution WRF model with boundary conditions taken from CORDEX RCM simulations (see Section 4.2) at 25 km resolution (Figure 1)—most likely the new CORE simulations (Coppola *et al*. 2021). The computational intensity of running WRF with BEP–BEM will limit the number of simulations that can be undertaken and hence the number of scenarios and policies that can be explored. The larger CORDEX ensembles available for Europe/Africa/East Asia (see Section 4.2), as well as the city-region projections (see Section 5), will, however, provide wider uncertainty ranges for these sensitivity studies and a baseline for assessing the added value of increasing resolution and complexity. This approach further illustrates the benefits of using a cascade of models, with the potential to eventually downscale to the building scale using, for example, the WRF cross-scale urban modelling system (Chen *et al*. 2011).

## 7 Conclusions: Climate Modelling and Urban Planning

Cities worldwide are already experiencing the impacts of human-induced climate change. Even with effective global action to reduce greenhouse gas (GHG) emissions, they will need to adapt to ongoing climate change, particularly rising temperatures and increased heat stress and heat-related mortality, as well as changes in heavy rainfall and drought. Effective policy and practice with the ambition of developing sustainable and healthy cities should include climate information as part of the evidence base. This paper illustrates how city stakeholders can use climate projections to better inform their decisions about how to effectively mitigate and adapt to climate change. These projections can be used to assess, for example, how future heat impacts might affect plans to increase active travel (walking and cycling). The examples of the climate projections being developed for the six Complex Urban Systems for Sustainability and Health (CUSSH) partner cities (see Section 2 and Table 1) illustrate the challenges and the approaches used to develop projections for urban areas.

A cascade of climate models of increasing complexity and spatial resolution (see Section 4.5 and Figure 1) provides the basis for constructing climate projections—from global climate models (GCMs) with a typical grid-box resolution of a few hundred kilometres (see Section 4.1), through regional climate models (RCMs) at 12–50 km (see Section 4.2) to convection-permitting models (CPMs) with a 1 km resolution (see Section 4.3). The most commonly implemented versions of these climate models do not include urban processes such as those which cause the urban heat island (UHI) (see Section 3). Increasingly, however, urban canopy models (UCMs)—again of varying complexity—are being incorporated into climate models (see Section 4.4). This gives the potential to explore how the UHI may evolve in individual cities against a backdrop of rising temperatures due to climate change—and how it may be modified by interventions such as greater use of green and/or blue infrastructures.

The city-region GCM-based projections produced for the CUSSH partner cities (see Section 5) indicate the benefits of global policies to reduce greenhouse emissions, and also provide the basis for qualitative risk assessment of the health impacts of climate change such as increasing heat stress. Ways in which these large-scale projections could be further tailored to explore issues such as spatial patterns of change across individual cities and heat stress and mortality at the neighbourhood level, as well as thermal comfort and energy use at the building scale, are also discussed (see Section 6). For CUSSH, planned modelling of specific cities, carried out as part of the partner Health and Economic impacts of Reducing Overheating in Cities (HEROIC) project, aims to provide evidence to policymakers as to the environmental, health, and health–economic costs and benefits of various city-scale interventions intended to reduce the health impacts of the UHI. This more detailed modelling work, particularly of the associated health impacts, will allow the effectiveness of proposed interventions to address the environmental and health priorities of the CUSSH partner cities (Table 1) to be evaluated and tracked. Thus, it will inform the implementation, as well as the identification, of appropriate urban policy measures.

Further work is also needed with respect to how these projections can be most effectively communicated and visualised for different CUSSH audiences. For some of these audiences, such as citizen focus groups, approaches such as storylines and narratives may be more appropriate than the maps and graphs conventionally used by climate scientists. Such narratives could be constructed for different levels of global warming (*e.g*. 1.5, 2°C; Lennard *et al*. 2018; Osima *et al*. 2018) rather than for specific emissions scenarios. A particular challenge is how best to convey appropriate information about modelling uncertainty and model performance, recognising that there is no such thing as a perfect climate model (Flato *et al*. 2013; Ogwang *et al*. 2016).

The CUSSH work could also be extended by considering heat stress indices which are more directly related to human thermal comfort and occupational performance than the meteorological indices presented in Section 5. Such indices include apparent temperature, humidex and simplified wet bulb temperature, as well as indices related to energy use such as cooling degree-days (Matthews 2018; Parkes *et al*. 2019). In order to fully address outdoor and indoor thermal comfort in urban areas, additional microscale effects may need to be considered, such as the impact of urban geometry on wind speed (Rajagopalan *et al*. 2014) and changes in urban solar radiation associated with changes in cloud cover (Theeuwes *et al*. 2019).

It is important that climate modellers understand and engage with the complex social, political, cultural and environmental factors affecting climate adaptation policies in cities, including historical policies that may have contributed to inequitable exposures to the UHI (Hoffman *et al*. 2020). These complex factors highlight the value of participatory engagement and an interdisciplinary environment for projects such as CUSSH (Davies *et al*. 2021). The CUSSH programme theory encompasses both action and change models, together with evaluation of processes and outcomes (Moore *et al*. 2021). In the action model, climate model information feeds into Step 6 (Build and use models), *i.e*. it supports and informs rather than determines policy in a bottom-up rather than a top-down approach.

While the CUSSH climate experts have not so far been directly involved in discussions with city stakeholders, they have gained a better understanding of what is useful and usable for cities through engagement with the CUSSH experts in health, governance and planning. The latter experts are talking directly to planners, policymakers and citizens in the partner cities. This learning process is recognised in the CUSSH theory of change model which encompasses changes in research and people (Moore *et al*. 2021). It is anticipated that the high-resolution (1 km) simulations planned for individual CUSSH cities (see Section 6) will provide opportunity for more targeted translation of findings and direct conversations between climate modellers and planners. These new dialogues should further help to improve the uptake of the science by policy and decision-makers.

In this context, this paper is framed with the ambition to better inform urban planners and policymakers about the different types and uses of climate models, thus addressing one of the identified barriers to evidence-based decision-making (Moore *et al*. 2021). With such improved understanding, users should be better placed to navigate and use effectively the growing array of climate adaptation tools^13^ and urban climate services^14^ that provide access to climate data and projections (Baklanov *et al*. 2020; Cortekar *et al*. 2016; Gidhagen *et al*. 2020; Schaeybroeck *et al*. 2020) and to participate in new communities of practice such as Future Earth’s Urban Knowledge Action Network.^15^ Through such activities, including involvement in projects such as CUSSH, planners, architects and engineers can better harness detailed climate modelling information and projections to inform their decisions about building morphology (shape) as well as the configurations of buildings and streets for shading and ventilation, albedo and materials, surface permeability (drainage), and green spaces.

The CUSSH research has highlighted the need to consider the uses and users of research, and this has implications for the researchers, including climate scientists, and research process. Different approaches to knowledge production and the relationships between ‘producers’ and ‘users’ are required at the science–policy interface. Understanding what works will contribute to more accessible and usable evidence for urban decision-makers.

## Supplementary Material

Supplementary data file 1

Supplementary data file 2

## Figures and Tables

**Table 1:** Summary characteristics of the six Complex Urban Systems for Sustainability and Health (CUSSH) partner cities.

	LONDON, UK	RENNES, FRANCE	KISUMU, KENYA	NAIROBI, KENYA	BEIJING, CHINA	NINGBO, CHINA
Latitude/ longitude (°)	51.5 N 0.1 W	48.1 N 1.7 W	0.1 S 34.8 E	1.3 S 36.8 E	39.9 N 116.4 E	29.9 N 121.6 E
Population (‘000)^a^	9,046	346	335	4,386	19,618	3,815
Area (km^2^)^b^	MUA: 1,046 Administrative: 1,612	MUA: 94 Administrative: 52	MUA: n.a. Administrative: 546	MUA: 562 Administrative: 713	MUA: 2,417 Administrative: 16,393	MUA: 1,123 Administrative: 8,917
Climate classification^c^	Cfb: Temperate, without a dry season, warm summer	Cfb: Temperate, without a dry season, warm summer	Af: Tropical, rainforest	Border of Cfb/Cwb: Temperate without a dry season/dry winter, warm summer; and Aw: Tropical, savannah	Bsk: Arid, steppe, cold bordering Dwa/Dwb: Cold, dry winter, hot/ warm summer	Csc: Temperate, dry winter, warm summer
Mean annual temperature (°C)^d^	10.3	11.6	22.9	19.0	13.1	16.4
Mean annual rainfall (mm)^d^	630	770	1,490	900	520	1,350
Authority responsible for urban planning	32 boroughs governed by the Greater London Authority (GLA). London Assembly led by the mayor	Rennes metropole has an intercommunal structure comprising 43 municipalities	County Government of Kisumu	Nairobi City County Government	People’s Government of Beijing Municipality	People’s Government of Ningbo Municipality
Key climate-related urban policies	London Plan. London Environmental Strategy	Rennes Plan for Climate Air and Energy	Kisumu County Environment Policy 2019	Nairobi Integrated Urban Development Master Plan	Plan for Healthy Beijing 2030	Plan for Healthy China 2030
Preliminary environmental and health priorities addressed in CUSSH	Greenhouse gas reduction. Green infrastructure. Active travel	Greenhouse gas reduction. Active travel	Waste management. Indoor air pollution (dirty fuels). Integrated spatial planning	Housing and sanitation. Indoor air pollution (dirty fuels). Spatial planning (decentralisation of functions to subcentres)	Air pollution. Heat stress. Mortality and morbidity	Air pollution. Heat stress. Mortality and morbidity

*Note:*

a In 2018, United Nations world urbanisation prospects (UNDESA 2018).

b Morphological urban areas (MUAs). Administrative area, The Global Administrative areas dataset (*https://gadm.org/*) (GAA 2012).

c Køppen Geiger classifications. High-resolution (5 arc min) Google Earth files download at: *http://koeppen-geiger.vu-wien.ac.at/present.htm*.

d These are calculated from CRU TSv3.26 (*https://crudata.uea.ac.uk/cru/data/hrg/*) for mean temperature; and the Global Precipitation Climatology Centre (GPCC) (*https://www.cgd.ucar.edu/cas/catalog/surface/precip/gpcc.html*) for precipitation total. Temperature indices of extremes are calculated from the Japanese Reanalysis (JRA-55) (*http://jra.kishou.go.jp/index.html*; and *https://www.jstage.jst.go.jp/article/jmsj/93/1/93_2015-001/_article*); and precipitation indices of extremes from the GPCC-FDD (full daily data) gridded data set (*ftp://ftp.dwd.de/pub/data/gpcc/html/fulldata-daily_v1_doi_download.html*).

**Table 2:** Projected changes (°C, % or days) in 30-year averages for Kisumu, Kenya, with respect to a present-day baseline 1981–2010, for the 2030s (2021–50), 2050s (2035–64) and 2080s (2071–2100).

KISUMU	OBSERVED	2030S: RCP8.5	2050S: RCP8.5	2080S: RCP8.5	2080S: RCP2.6
Mean temperature (°C)	22.9	+1.3 (0.8 to 1.8)	+1.9 (1.2 to 2.6)	+3.9 (2.7 to 5.1)	+1.1 (0.3 to 1.8)
Warm days	33% of days	+28 (11–42)%	+38 (19–53)%	+60 (43–73)%	+24 (12–37)%
Warm nights	31% of days	+44 (29–58)%	+59 (42–73)%	+80 (67–85)%	+38 (25–54)%
Total rainfall	1,490 mm	+8 (–3 to +27)%	+12 (–4 to +33)%	+29 (+2 to +76)%	+7 (–9 to +22)%
Heavy rainfall days	47	+7 (–2 to +20)	+9 (–3 to +28)	+23 (–1 to +65)	+7 (–3 to +23)
Consecutive dry days	17	0 (–3 to +4)	0 (–3 to +4)	0 (–6 to +9)	0 (–4 to +4)

*Note:* The average change is shown together with an indication of the uncertainty range across the models (in parentheses = 90% probability range). Since the changes for RCP2.6 and RCP8.5 are similar until mid-century, RCP2.6 changes are shown only for the 2080s (final column). The observed values are grid-point averages^a^—such values will always differ somewhat from values for a single station. See Table S1 in the supplemental data online for definitions of the climate indices.

a These are calculated from CRU TSv3.26 (*https://crudata.uea.ac.uk/cru/data/hrg/*) for mean temperature; and the Global Precipitation Climatology Centre (GPCC) (*https://www.cgd.ucar.edu/cas/catalog/surface/precip/gpcc.html*) for precipitation total. Temperature indices of extremes are calculated from the Japanese Reanalysis (JRA-55) (*http://jra.kishou.go.jp/index.html*; and *https://www.jstage.jst.go.jp/article/jmsj/93/1/93_2015-001/_article*); and precipitation indices of extremes from the GPCC-FDD (full daily data) gridded data set (*ftp://ftp.dwd.de/pub/data/gpcc/html/fulldata-daily_v1_doi_download.html*).

## Data Availability

Data for the GCM-based projections (see Section 5 and File 2 in the supplemental data online) are provided via the CUSSH data pages: *https://www.ucl.ac.uk/complex-urban-systems/data*.

## Footnotes

1 For the WHO’s Healthy Cities movement definition of a healthy city, see *https://www.who.int/healthy_settings/types/cities/en/*.

2 See *https://www.ucl.ac.uk/complex-urban-systems/*.

3 See *https://climate-adapt.eea.europa.eu/knowledge/tools/urban-ast/step-2-0/*.

4 Climate Resilient Cities and Infrastructure (RESIN), *https://resin-cities.eu/*.

5 See *https://cordex.org/*.

6 See *https://climate-adapt.eea.europa.eu/knowledge/tools/urban-adaptation*.

7 See *https://www.wudapt.org/*.

8 See *https://www.who.int/activities/monitoring-health-impacts-of-climate-change-and-national-progress/*.

9 These are calculated from CRU TSv3.26 (*https://crudata.uea.ac.uk/cru/data/hrg/*) for mean temperature; and the Global Precipitation Climatology Centre (GPCC) (*https://www.cgd.ucar.edu/cas/catalog/surface/precip/gpcc.html*) for precipitation total. Temperature indices of extremes are calculated from the Japanese Reanalysis (JRA-55) (*http://jra.kishou.go.jp/index.html*; and *https://www.jstage.jst.go.jp/article/jmsj/93/1/93_2015-001/_article*); and precipitation indices of extremes from the GPCC-FDD (full daily data) gridded data set (*ftp://ftp.dwd.de/pub/data/gpcc/html/fulldata-daily_v1_doi_download.html*).

10 See *https://rdrr.io/github/GHI-UW/HOT/*.

11 See *https://iiasa.ac.at/web/home/research/researchPrograms/air/GAINS.html/*.

12 Wellcome Trust HEROIC project (grant number 216035/Z/19/Z), *https://www.ucl.ac.uk/bartlett/environmental-design/research-projects/2020/dec/heroic-health-and-economic-impacts-reducing-overheating-cities/*.

13 See *https://climate-adapt.eea.europa.eu/knowledge/tools/urban-ast/step-2-0/*; and *https://climate-adapt.eea.europa.eu/knowledge/tools/urban-adaptation*.

14 Examples of urban climate services development in Europe include: *https://www.urban-climate.eu/*; *https://www.ukclimateresilience.org/projects/climate-service-pilots/*; *https://dataplatform.climate-fit.city/*; and *http://urclim.prod.lamp.cnrs.fr/*.

15 See *https://futureearth.org/networks/knowledge-action-networks/urban/*.
