# Supplementary material for: Climate change projections for sustainable and healthy cities
Source: Build Cities. Author manuscript; Available in PMC 2021 Oct 25. (PMC7611885; doi:10.5334/bc.111)
Supplement: Supplementary data file 1 [file EMS137020-supplement-Supplementary_data_file_1.pdf]

The urban canopy and boundary layers

The UHI can be split into two vertical layers (Oke 1976; Oke 1978; Oke et al. 2017).

The lower layer is the **urban canopy layer**, the layer at building level caused by the roughness of the urban surface. This is dominated by the urban surface and will be different depending on the material and form of the surrounding environment, for example an area dense with buildings versus a park. This layer is produced by microscale processes (Figure 1).

The upper layer is the **urban boundary layer**, a local to mesoscale concept (Figure 1). This is the area of the planetary boundary layer where the presence of the city has modified the climatic characteristics. The increased roughness of the city can cause local slowing of wind over the city. This causes convergence of air and can cause the boundary layer to 'dome' up over the city. For potentially tens of kilometres downwind of the city, an urban plume of rising air can occur.

Contributory causes of the UHI

Six factors which affect the surface energy balance of a city and thus are contributory causes of the UHI have been identified (Howard 1833; Oke 1981). These are listed below and discussed briefly in terms of their influence on the surface energy balance. This energy balance can be expressed as:

$$Q^* + Q_F = Q_E + Q_H + \Delta Q_S$$

where Q^* is the net surface radiant flux density, Q_F the anthropogenic heat flux density, Q_E the latent heat flux density, Q_H the sensible heat flux density and Q_S is the heat stored (Oke 1982).

- Anthropogenic heat (Q_F)
  - Heat is generated within the city from three main sources: vehicle emissions, buildings, and metabolic heat of people (Allen et al. 2011)
- Impervious surfaces
  - Moisture availability is required for latent heat loss (Oke 1982) and tends to be lower over impervious city surfaces such as asphalt, concrete and metal and where drainage systems remove water, than over soil and vegetation such as in city parks. In cities the main energy release tends to come from sensible heating rather than latent energy (Oke et al. 1992).
- Thermal properties of the city fabric
  - City materials tend to have higher thermal conductivity and heat capacity than surrounding areas. Thus Q_S is generally larger in cities. The properties of these materials also mean that they release stored heat relatively easily, thus playing a major role in the nocturnal UHI (Oke 1982).
- Surface geometry
  - Urban street canyons prevent the radiation of heat back to the sky in all directions and the building walls also tend to absorb heat, then radiate it back

to the surface (Oke et al. 1991). Thus dense cities with tall buildings cool at night much slower than surrounding rural areas (Oke 1981).

- Urban roughness
  - Tall inflexible buildings are associated with higher roughness parameters than the more flexible and softer vegetation in surrounding rural areas. Air decelerates as it flows over higher roughness areas, limiting the dispersal of heat and pollution generated by the city (Barlag & Kuttler 1990). A roughness sublayer can form, up to several times the average building height in extent and consisting of interactive waves and plumes of heat, humidity and pollutants.
- Air pollution
  - Increased levels of aerosols from air pollution in urban areas are expected to absorb, scatter and reflect incoming short-wave radiation and emit it as long-wave radiation. Thus polluted urban areas should receive less solar radiation (Li et al. 2018; Oke 1978). In practice, these differences tend to be partially offset by the typically lower albedo of urban areas.

References

Allen, L., Lindberg, F., & Grimmond, C. S. B. (2011). Global to city scale urban anthropogenic heat flux: Model and variability. *International Journal of Climatology*, *31*(13), 1990–2005. DOI: https://doi.org/10.1002/joc.2210

Barlag, A. B., & Kuttler, W. (1990). The significance of country breezes for urban planning. *Energy and Buildings*, *15*(3–4), 291–297. DOI: https://doi.org/10.1016/0378-7788(90)90001-Y

Howard, L. (1833). *THE CLIMATE OF LONDON*.

Li, H., Meier, F., Lee, X., Chakraborty, T., Liu, J., Schaap, M., & Sodoudi, S. (2018). Interaction between urban heat island and urban pollution island during summer in Berlin. *Science of the Total Environment*, *636*, 818–828. DOI: https://doi.org/10.1016/j.scitotenv.2018.04.254

Oke, T. R. (1981). Canyon Geometry and the Urban Heat Island. *Journal of Climatology*, *1*, 237–254.

Oke, T. R., Zeuner, G., & Jauregui, E. (1992). The surface energy balance in Mexico City. In *Atmospheric Environment* (Vol. 26, Issue 4).

Oke, T. R. (1978). *Boundary Layer Climates* (2nd ed., pp. 273–294). Methuen & C, Routledge.

Oke, T. R. (1982). The energetic basis of the urban heat island. *Quarterly Journal of the Royal Meteorological Society*, *108*(455), 1–24. DOI: https://doi.org/10.1002/qj.49710845502

Oke, T. R. (1976). The distinction between canopy and boundary-layer urban heat Islands. *Atmosphere*, *14*(4), 268–277. DOI: https://doi.org/10.1080/00046973.1976.9648422

Oke, T. R., Johnson, G. T., Steyn, D. G., & Watson, I. D. (1991). Simulation of surface urban heat islands under “ideal” conditions at night part 2: Diagnosis of causation. In *Boundary-Layer Meteorology* (Vol. 56, Issue 4). DOI: https://doi.org/10.1007/BF00119211

Oke, T. R., Mills, G., Christen, A., & Voogt, J. A. (2017). Urban climates. In *Urban Climates*. Cambridge University Press. DOI: https://doi.org/10.1017/9781139016476
