# Supplementary material for: Climate change projections for sustainable and healthy cities
Source: Build Cities. Author manuscript; Available in PMC 2021 Oct 25. (PMC7611885; doi:10.5334/bc.111)
Supplement: Supplementary data file 2 [file EMS137020-supplement-Supplementary_data_file_2.pdf]

## City-region climate projection data for the CUSSH partner cities

In order to provide climate projection data for the regions containing each of the CUSSH partner cities (London, Rennes, Kisumu, Nairobi, Beijing, Ningbo) during the early stages of the project, use was made of large pre-existing data set. This document describes the city-region projection data provided to CUSSH colleagues in February 2019 and included in Goodess et al., 2021, Climate change projections for sustainable and healthy cities, *Buildings and Cities*.

The starting point for producing these projections was the post-processed CMIP5 ensemble of global climate model (GCM) runs that we employed for production of country profiles for the WHO (https://www.who.int/activities/monitoring-health-impacts-of-climate-change-and-national-progress). The original data set produced for the WHO included annual mean temperature and precipitation totals (and derived indices of maximum/minimum temperature and precipitation extremes calculated from daily data) and absolute humidity, for two emissions scenarios: RCP2.6 and RCP8.5. One of the main advantages of the CMIP5 data is the relatively large ensemble size (important for assessing climate modelling uncertainty – we worked with about 20 GCMs for the WHO analysis) and the availability of an emissions scenario consistent with the 2˚C Paris policy target (RCP2.6) as well as a higher business-as-usual scenario (RCP8.5). For CUSSH, an important characteristic of these data from GCMs and the accompanying gridded observations is that the information is consistent across cities. The disadvantage is the relatively coarse spatial scale.

For the WHO work, we also included indices based on gridded observations.  For the profiles published in 2019 and the CUSSH work we calculated temperature indices of extremes from the Japanese Reanalysis (JRA-55[1]) and precipitation indices of extremes from the GPCC-FDD (Global Precipitation Climate Centre – Full Daily Data) gridded data set[2]. Mean temperature and total annual precipitation use CRU-TS-v3.26[3] and GPCC[4] respectively. The gridded observations are used to apply a simple bias adjustment to the simulated indices (Figure 1).

As Figure 1 shows, the methodology developed for the WHO for producing country averages involves interpolating all data sets to a common 0.5 degree latitude/longitude grid. From this common grid it is possible to extract data for the individual grid boxes in which specific cities are located.

Data were extracted for all six CUSSH case study cities. As well as summary time-series plots (.pdf files in **plots_offset**), summary statistics (in a series of .csv files which can be readily opened in Excel for example) are also provided (absolute average values in **abs_summaries_csv**) and changes in **delta_summaries_csv** for 30-year time periods: 1961-1990, 1971-2000, 1981-2010 (recommended baseline for projections), 2021-2050, 2035-2064 and 2071-2100). The time series plots show indices for a high emissions scenario, Representative Concentration Pathway 8.5 (RCP8.5 - in orange) and a low emissions scenarios (RCP2.6 – in green). The figures also show each model individually as well as the 90% model range (shaded) as a measure of uncertainty and the annual and smoothed observed record (in blue). The underlying data are provided in the accompanying .xlsx data files in

[1] http://jra.kishou.go.jp/index.html and https://www.jstage.jst.go.jp/article/jmsj/93/1/93_2015-001/_article
[2] ftp://ftp.dwd.de/pub/data/gpcc/html/fulldata-daily_v1_doi_download.html
[3] https://crudata.uea.ac.uk/cru/data/hrg/
[4] https://www.cgd.ucar.edu/cas/catalog/surface/precip/gpcc.html

**excel_files_offset** (note that the data files contain multiple data sheets and all values are unsmoothed).

Six indices of temperature extremes (TN90p, TN10p, TX90p, TX10p, CSDI and WSDI) and four indices of precipitation extremes (R10mm, R20mm, CDD and R95pTOT) are provided (Table 1). These indices are a subset of the 27 indices recommended by the ETCDDI (Expert Team on Climate Change and Detection Indices) and CLIMDEX (https://www.climdex.org/index.html). All indices are calculated annually using daily data and a baseline period of 1961-1990.

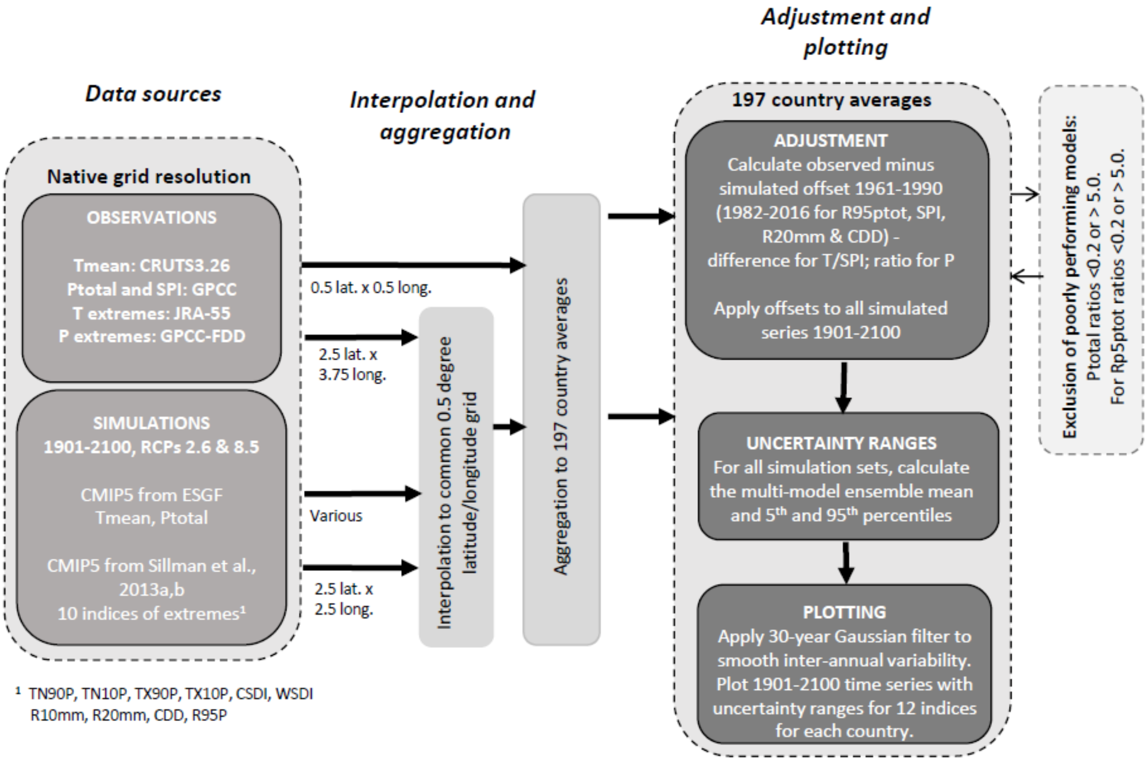


Figure 1: Methodological steps for processing observed/modelled data for the WHO climate and health country profiles published in 2019.

**Table 1: Definition and description of the indices available for the CUSSH city-region projections. TN: minimum temperature; TX maximum temperature.**

| Index code | Descriptive name | Definition | Units |
|---|---|---|---|
| **Tmean** | Mean temperature | Mean annual temperature | °C |
| **Tmax** | Mean maximum temperature | Mean annual maximum temperature | °C |
| **Tmin** | Mean minimum temperature | Mean annual minimum temperature | °C |
| **Ptotal** | Precipitation total | Annual precipitation total | mm |
| **TN90p** | Warm nights | Percentage of days when TN > 90$^{th}$ percentile | % of days |
| **TN10p** | Cold nights | Percentage of days when TN < 10$^{th}$ percentile | % of days |
| **TX90p** | Warm days | Percentage of days when TX > 90$^{th}$ percentile | % of days |
| **TX10p** | Cold days | Percentage of days when TX < 10$^{th}$ percentile | % of days |
| **CSDI** | Cold Spell Duration Indicator | Annual count of days with at least 6 consecutive days when TN < 10$^{th}$ percentile | Days |
| **WSDI** | Warm Spell Duration Indicator | Annual count of days with at least 6 consecutive days when TX > 90$^{th}$ percentile | Days |
| **R10mm** | Number of heavy precipitation days | Annual count of days when precipitation ≥ 10 mm | Days |
| **R20mm** | Number of very heavy precipitation days | Annual count of days when precipitation ≥ 20 mm | Days |
| **CDD** | Consecutive dry days | Maximum number of consecutive days with no precipitation (precipitation < 1mm) | Days |
| **R95pTOT** | Very wet days contribution | Contribution to annual total precipitation from days when precipitation >95$^{th}$ percentile (very wet days) | % |
